# Correlation study: electrical impedance-based approximation of knee joint angle and extensor strength during concentric knee extension

**DOI:** 10.1186/s12891-026-09634-y

**Published:** 2026-02-18

**Authors:** Jacob P. Thönes, Franziska Geiger, Judith Osterloh, Arash Keshavarz, Rainer Bader, Sascha Spors

**Affiliations:** 1 Institute of Communications Engineering, Rostock, Germany; 2 Department of Orthopaedics, Rostock University Medical Center, Rostock, Germany

**Keywords:** Impedance measurements, Muscle function

## Abstract

Musculoskeletal disorders often lead to pain, limited mobility, and reduced quality of life. Monitoring muscle function is crucial for understanding and treating these conditions, yet current methods like dynamometry are impractical for daily use. Consequently, there is a clear need for practical, clinically applicable solutions to estimate muscle function. This work used electrical impedance tomography (EIT) measurements as a non-invasive alternative. EIT data were recorded from the thigh during concentric knee extension at predefined torque levels, simultaneously measured with a dynamometer. A data-driven model was applied to analyze conductivity changes linked to muscle activation. Results indicate that EIT signals reflect knee joint angle and extensor strength, highlighting its potential for portable, real-time monitoring in clinical settings.

## Introduction

Musculoskeletal disorders affecting bones, joints, and muscles, such as osteoarthritis, rheumatoid arthritis, and osteoporosis, have become increasingly relevant nowadays. These diseases often result in chronic pain, restricted mobility, and functional impairments, significantly reducing the quality of life [1]. The measurement of muscle strength plays an important role in understanding and treating these disorders. It provides insights into muscle functionality, response to loads, and recovery processes [2, 3]. Knee extensor strength is a key determinant of knee pain, functional capacity, and fall risk across a wide range of musculoskeletal disorders, making its assessment essential for the evaluation of musculoskeletal function and clinically relevant outcomes [4, 5]. Technology-based assessments for self-monitoring help individuals to reflect on their self-awareness of their own behaviours, thereby promoting better adherence to treatment [6, 7]. Isokinetic dynamometry remains one of the most frequently used methods for assessing muscle strength. However, its use is limited by the size and weight of the measurement devices, making it unsuitable for self-monitoring in daily life. Furthermore, several non-invasive approaches are currently used for the assessment of muscle strength in a clinical setting. This includes manual muscle testing. It is widely employed in clinical practice due to its simplicity and minimal equipment requirements. Hand-held dynamometry provides more objective measurements while remaining relatively portable. These methods rely on subjective evaluation or require externally applied force and user cooperation, limiting their suitability for continuous, unobtrusive monitoring outside controlled environments. In this context, electrical impedance tomography (EIT) represents an alternative modality for muscle strength assessment.

EIT is a technique that can be used to reconstruct spatial impedance images from surface measurements [8]. In clinical practice, it is an established method for respiratory monitoring [9]. Small alternating currents are injected through surface electrodes while recording the resulting boundary voltages on the remaining electrodes. These voltages are linked to the conductivity distribution within the imaged cross-section. This cost-effective, non-invasive, and radiation-free method is particularly suited for the development of portable technologies due to its energy-efficient and simple design [10]. The measured distribution of electrical impedance within an object can be used to infer tissue composition and functional status [8, 11]. Recent studies demonstrated that EIT can be used for estimating musculoskeletal joint kinematics, such as wrist angle [12], wrist motion [13], and hand gesture estimation [14]. Li and Lee proposed a wrist-worn joint torque estimation method using voltage-torque mapping and machine learning on electrical impedance signals [15]. Schouten et al. combined multifrequency electrical impedance myography/tomography with surface EMG to jointly estimate joint angle and torque, demonstrating the benefit of multimodal sensing and neural network models [16]. More recently, Zheng et al. employed an EIT-based musculoskeletal model to represent human arm dynamic intent for human robot interaction, enabling more expressive control strategies [17]. Advances in learning-based modeling have further improved force and rehabilitation related applications. Zhang et al. introduced a transformer-based model with spatial–frequency feature fusion for muscle force estimation using EIT measurements [18]. Liang et al. proposed EIT for monitoring knee angle and muscle strength after total knee arthroplasty [19]. Hence, EIT has the potential to provide a non-invasive, indirect assessment of muscle strength and function. However, as reviewed, all articles, except the work from Liang et al. [19], focused on arm, wrist, or hand estimation.

Based on these findings, we hypothesize that EIT, combined with a machine learning approach, may be used to estimate dynamic knee joint angle and knee extensor strength during concentric knee extension. For the first proof of concept, EIT data were recorded during a concentric knee extension with submaximal torque targets. Assessing dynamic muscle strength across multiple torque targets provides a more comprehensive evaluation of muscle health than isometric maximal force measurements, as it better reflects muscle force production and modulation during daily activities [20, 21]. Electrodes were placed on the skin of the participant’s thigh to measure the electrical impedance of the thigh muscles. At the same time, an isokinetic dynamometer recorded the torque generated during the concentric knee extension. Variations in electrical conductivity, influenced by tissue properties and functional states of the muscle, were evaluated by different machine learning models. This approach could provide a non-invasive, portable, and practical method for real-time monitoring of muscle forces, offering significant potential for later clinical rehabilitation. This work aims to contribute to bridging the gap between advanced imaging techniques and the practical needs of monitoring the health functionality of the musculoskeletal system.

## Methodology

### Participants

Fifteen healthy participants (ten females and five males) aged between 20 and 30 years (24.73 ± 4.32 years) took part in the study, with the majority exhibiting right-leg dominance (13 out of 15) according to the Waterloo Footedness Questionnaire-Revised [22]. Prior to data collection, all participants were thoroughly briefed regarding the study’s objectives, experimental procedures, and their rights as participants, and gave their informed consent. Ethical approval was granted by the Ethics Committee of the Rostock University Medical Center, Germany (A 2025-0016) before the initiation of the study. All methods were carried out in accordance with the relevant guidelines and regulations of the Declaration of Helsinki.

### Measurement setup

**Fig. 1:**
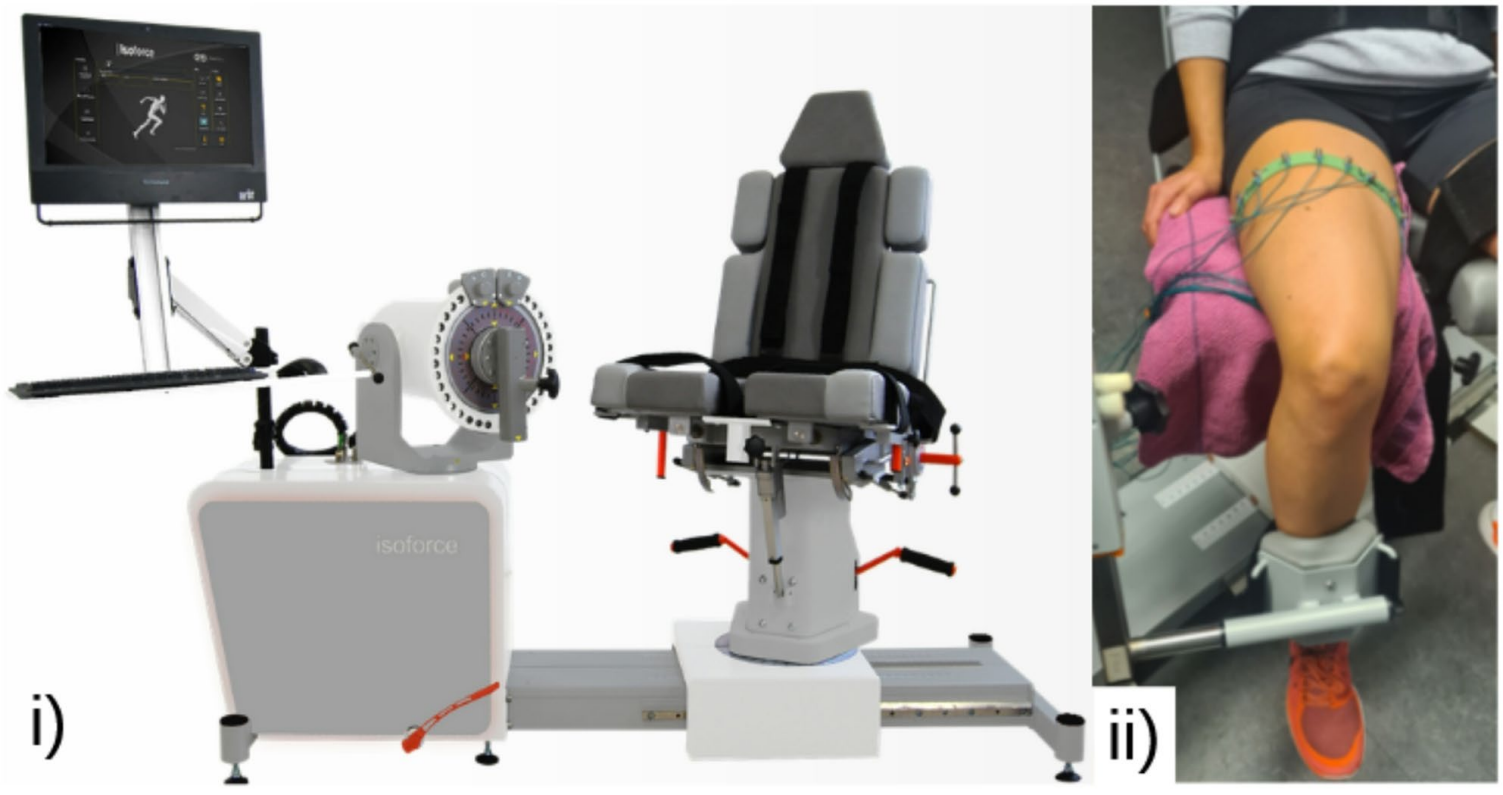
Measurement setup with (**i**) Isoforce™ (TUR GmbH, Berlin, Germany) for measuring the torque and controlling the movement (picture taken from: https://www.turweb.com/products/isoforce-1, 2025) and (**ii**) the leg of a participant with the self-made EIT belt for impedance data measurement.)

The experimental setup used two measurement devices: A Sciospec (Scientific Instruments GmbH, Bennewitz, Germany) EIT device and the isokinetic dynamometer torque measurement device Isoforce™ (TUR GmbH, Berlin, Germany) Fig. 1i). Both devices were sampled concurrently to ensure time aligned data acquisition, enabling accurate input-output mapping, for subsequent evaluation modeling. A custom-developed graphical user interface (GUI) was employed to coordinate signal acquisition, streamline metadata export, and support reliable data recording.

A custom-made elastic belt with 16 evenly spaced stainless steel screw electrodes was used for EIT measurements. The impedance data consisted of surface voltage measurements acquired during current-driven injection, covering all injection combinations over one full rotation using the surface electrodes. This resulted in 16 × 16 measurements per EIT sample. The belt was placed around the mid-thigh of the participant’s dominant leg and connected to the EIT device (Fig. 1ii). To improve comfort and reduce pressure from the electrodes during contractions, a folded towel was placed between the seat of the Isoforce™ and the electrode belt on the thigh. To ensure consistent electrode placement across all participants, one electrode was marked for orientation and was always placed in approximately the same position. The EIT device measurement parameters are summarized in Table 1.

**Table 1:** Configuration setup for the EIT measurement sciospec device (Scientific instruments GmbH, Bennewitz, Germany)

Parameter	Value
Excitation Frequency	125 kHz
Number of Electrodes	16
Injection Skip	5
Amplitude	1 mA
Frame Rate	40 Hz

Isokinetic torque measurements were performed using the Isoforce™ system. Each participant was positioned based on individual anthropometrics and range of motion. In order to estimate knee extensor strength from the measured torque, the force acting on the cuff of the dynamometer was determined. For this purpose, the subject-specific lever arm length $\:{d}_{l}^{\left(p\right)}\:$with superscript $\:\left(p\right)$ indicating the participant, was measured as the distance between rotation axis of the dynamometer which was aligned with the knee joint axis and the center of the dynamometer cuff. Each participant was instructed to extend their dominant leg from full flexion to full extension. To ensure a controlled concentric knee extension, a constant angular velocity of 30° s^− 1^ was set. For concentric knee extensor torque measurements 60° s^− 1^ [23] seems optimal, but to make it easier for the participants to quickly reach the target torque level and keep it steady during the movement, a slower pace was necessary. The participants were asked to track a visually presented target torque and maintain it throughout the entire movement (approx. 3 s). The lower leg was then passively returned to the initial position at an angular velocity of 15° s^− 1^.

To familiarize participants with the task, warm-up trials at torque levels of 20 Nm, 40 Nm, and 60 Nm were conducted. The main experimental session included fourteen trials across torque levels ranging from 20 Nm to 80 Nm, in 10 Nm increments. This target torque range was selected because it could be sustained by the majority of participants across a large proportion of the individual range of motion. Each torque level was recorded in two consecutive trials and in randomized order to minimize systematic fatigue effects, with approximately 30 s of rest between trials.

### Data recording and processing

**Fig. 2:**
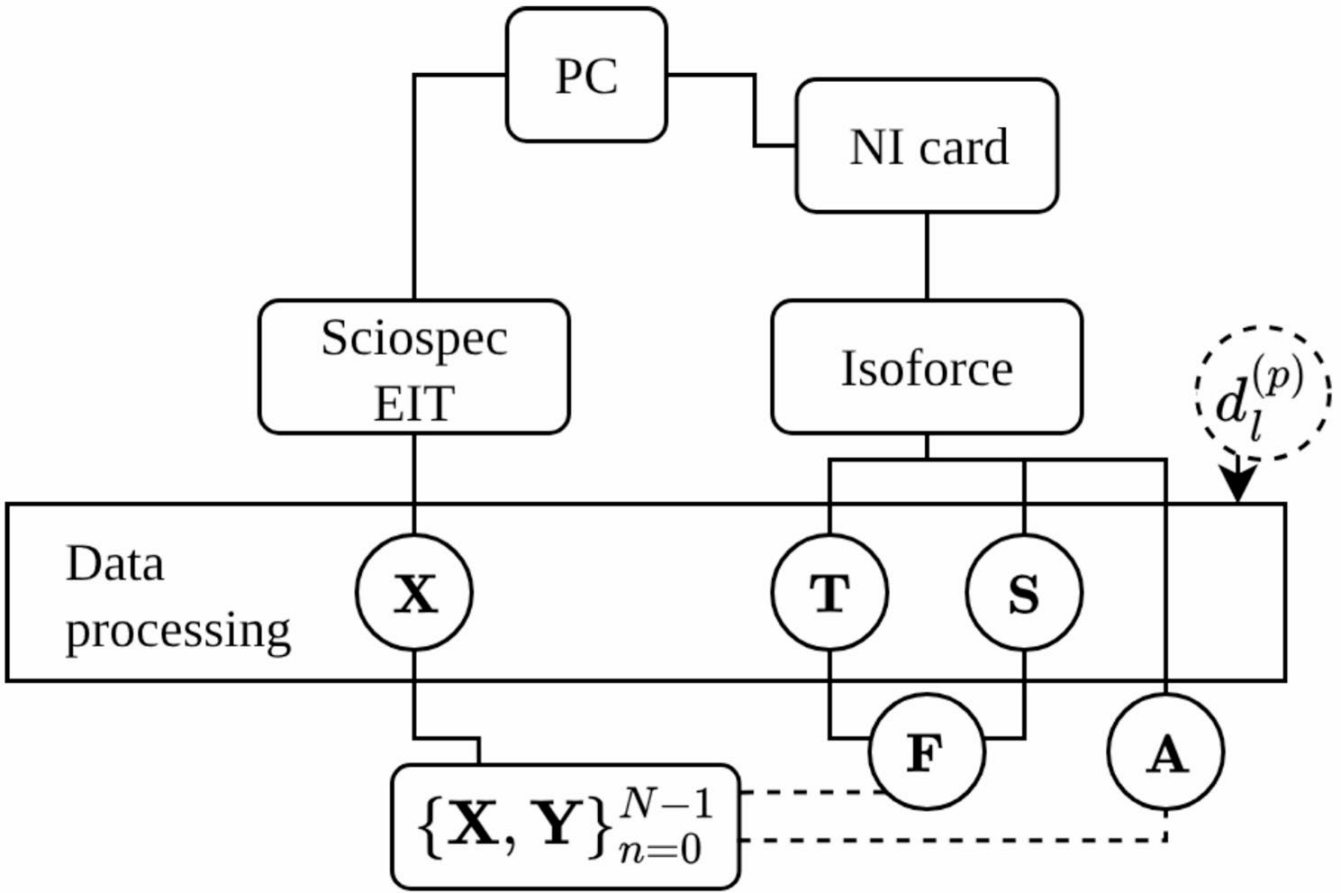
Recording pipeline for acquiring the impedance and Isoforce™ data. The participant-specific $\:\left(\boldsymbol{p}\right)$ lever arm length $\:{\boldsymbol{d}}_{\boldsymbol{l}}^{\left(\boldsymbol{p}\right)}\:$ was measured once during the calibration of the Isoforce™. The final processed data are the impedance measurements **X**, and the corresponding force $\mathbf{F}$ and angles **A**

The data recording is illustrated in Fig. 2. The Isoforce™ recorded the joint angle, torque, and speed at a sampling rate of 400 Hz without a time stamp. To enable later synchronization, the analog signals of the Isoforce™ were simultaneously recorded with a data acquisition (DAQ) system from National Instruments (NI). The analog torque (**T**), speed (**S**), and position angles (**A**) were captured at a sampling rate of 100 Hz. EIT impedance data (**X**) were recorded at 40 Hz. All recordings were subsequently checked for completeness. Due to differences in the sampling rates, a synchronization using SciPy (v1.15.2) between the Sciospec and Isoforce™ data was applied.

To determine the knee extensor strength ($\mathbf{F}$), the torque values were divided by the lever arm length $\:{d}_{l}^{\left(p\right)}\:$of each participant $\:\left(p\right)$.

The knee extensor strength labels were stored together with the corresponding impedance measurements in the dataset $\:{\left\{\mathbf{X},\:\mathbf{F}\right\}}_{n=0}^{N-1}$, with in total 67,615 data samples. A second dataset $\:{\left\{\mathbf{X},\:\mathbf{A}\right\}}_{n=0}^{N-1}\:$was prepared with the same sample size, comprising the impedance measurements with the corresponding angular lever arm positions representing the knee joint angle.

### Modal training

Three models were employed to evaluate different classification approaches and their performance. Support vector machines (SVMs), Random Forest (RF), and Logistic Regression (LR) classifiers. The SVM classifier aims to find an optimal separating hyperplane by implicitly mapping non-linear input data to high-dimensional feature spaces using different kernel functions [24]. RF is an ensemble-based classification method consisting of multiple decision tree classifiers [25, 26]. The aim of this algorithm is to construct a collection of trees whose individual predictions exhibit low mutual correlation, thereby enhancing the overall predictive performance of the ensemble [27]. LR estimates class probabilities using a logistic sigmoid function and weights linear combinations of input features [28]. Regularization can be applied to prevent the LR model from overfitting and improve generalization.

Hyperparameter optimization was performed using grid search for all models. For the RF classifier, the tuned parameters were the number of trees (*n_estimators*), maximum tree depth (*max_depth*), minimum number of samples required to split a node (*min_samples_split*), minimum number of samples per leaf (*min_samples_leaf*), and the number of features considered at each split (*max_features*). For the SVM, tuning included the kernel type (*kernel*), the regularization parameter (*C*), the kernel coefficient (*gamma*), and, for polynomial kernels, the polynomial degree (*degree*). For LR, the tuned hyperparameters comprised the regularization type (*penalty*), the inverse regularization strength (*C*), the optimization solver (*solver*), the regularization ratio (*l1_ratio*), and the use of class weighting (*class_weight*). All models were trained, validated, and tested. The validation is defined as a five-fold training and validation on all available data samples. The testing is defined as a five-fold participant-wise train-test split. Testing is more challenging for the models because the test data only includes measurements from unknown participants. The full pipeline of the models was implemented in Python using scikit-learn (version 1.7.1 [29]). The full code is available on GitHub [30]. We evaluated the ability of the mentioned model architectures to predict the knee extensor strength $\mathbf{F}$ from the impedance measurements $\mathbf{X}$. To visualize the performance, a confusion matrix plot was used. The confusion matrix indicates the model’s ability to accurately predict the correct classes. The accuracy score was computed as a metric for correctly classified samples. Accuracy is defined as the ratio of the number of true predictions to the total number of predictions. In scikit-learn (version 1.7.1 [29]), this metric returns a value between 0 and 1. Furthermore, we evaluated the ability to predict the knee joint angle $\mathbf{A}$ from impedance measurements $\mathbf{X}$. Due to the concentric knee movement, we expect the angle to have a significant impact on the impedance measurements. To evaluate this impact, we trained the best-performing model to predict knee joint angles at discrete knee extensor strength values.

## Results

**Fig. 3:**
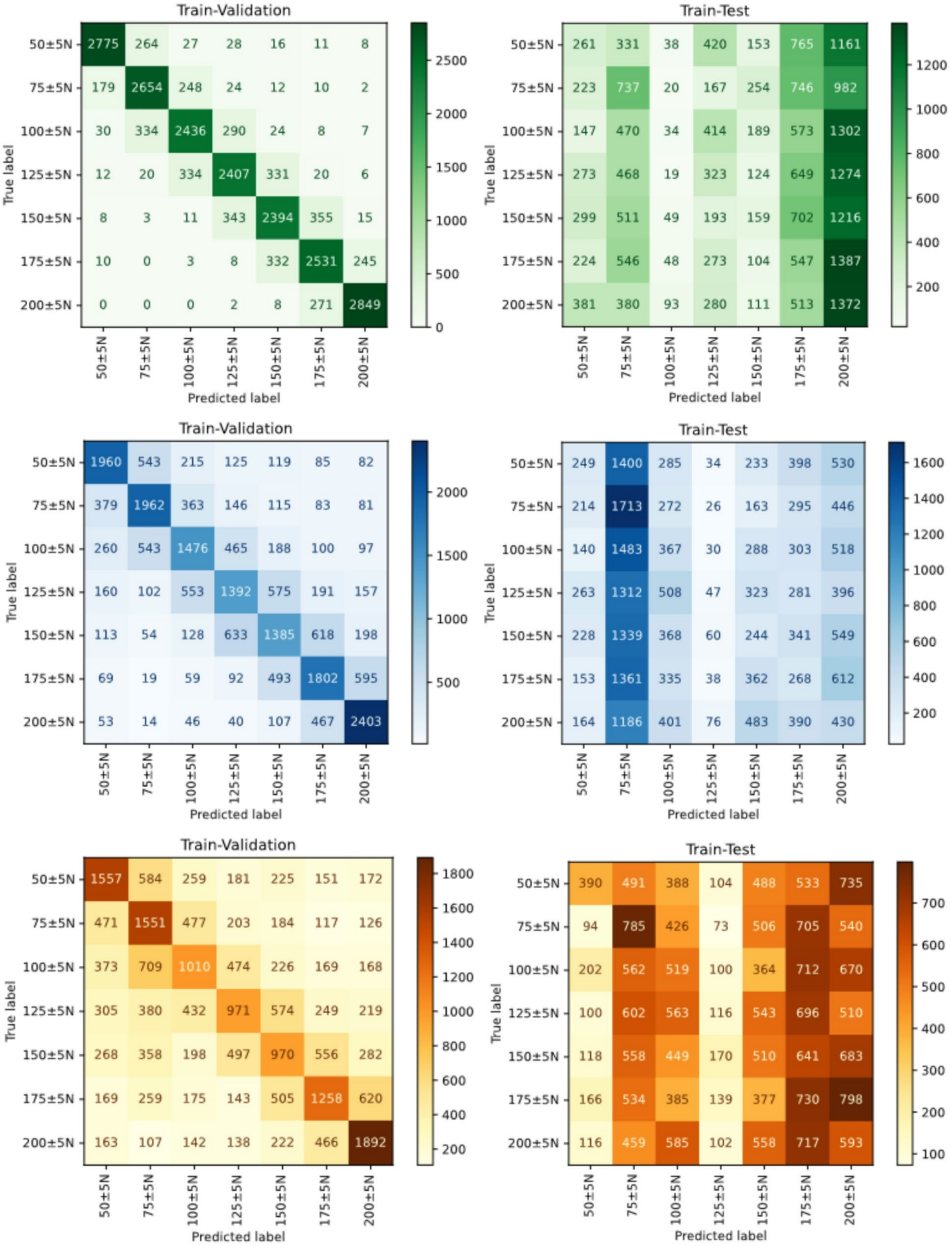
Visualization of the combined results from the five-fold train and test classifications for three force labels. The left column contains the combined results from the validation, and the right column contains the combined results from the test

The hyperparameter tuning of the models was done with a five-fold cross validation for evaluating the knee extensor strength $\mathbf{F}$ from the impedance measurements $\mathbf{X}$. The strength labels were categorized into seven classes, comprising all samples with the strength levels 50 N to 200 N, in 25 N steps, including a tolerance of ± 5 N. The best hyperparameters were saved, and the tuned models were reinitialized to produce the five-fold validation and participant-wise test results. The confusion matrices are visualized in Fig. 3. The fold-wise validation and test balanced accuracy score for RF, SVM, and LR are presented in Table 2. The balanced accuracy score was used to account for potential class imbalance. The balanced accuracy was computed as the arithmetic mean of the recall values obtained for each class.

**Table 2:** Balanced accuracy across folds for each models validation and test

Balanced accuracy score						
	RF		SVM		LR	
Fold	Valid	Test	Valid	Test	Valid	Test
1	0.831	0.151	0.569	0.169	0.425	0.159
2	0.827	0.179	0.569	0.171	0.417	0.165
3	0.820	0.192	0.562	0.169	0.421	0.195
4	0.823	0.144	0.562	0.118	0.416	0.127
5	0.817	0.128	0.564	0.142	0.423	0.164
Mean	0.824	0.157	0.565	0.151	0.420	0.166

On the validation data, RF consistently achieved the highest balanced accuracy across all folds, with a mean balanced accuracy of 0.824. This indicates strong fitting capacity and stable performance during cross-validation. SVM and LR showed balanced moderate discriminative ability, but noticeably lower than that of RF. In contrast, on the test data, all models exhibited a substantial drop in balanced accuracy. RF test performance was 0.157, while SVM and LR achieved comparable bad results. Across all folds, no single model demonstrated a clear and consistent advantage on the test set. The variability between folds was moderate, indicating no extreme instability.

Table 3 reports the RF performance for knee extensor strength estimation when the joint angle is held fixed. Specifically, the RF model was trained and evaluated to discriminate the seven discrete knee extensor strength targets again. Separate evaluations were conducted for four constrained angle conditions in 20° steps from 20° to 80°. Across all fixed-angle conditions, the RF achieved high balanced accuracies, with mean accuracies ranging from 0.897 to 0.939. The highest mean performance was observed at 60 ± 1° (mean balanced accuracy = 0.939), followed closely by 20 ± 1° (0.930). The relatively small variance across folds indicates stable knee extensor strength discrimination when the angular configuration is controlled, suggesting that strength-related signal features remain highly separable within narrow angular ranges. In contrast, Table 4 presents the complementary evaluation in which the RF model performs knee joint angle classification while the applied knee extensor strength is fixed. Here, four discrete angle targets (20° to 80° in 20° steps, including all samples within ± 1°) were classified separately for the same seven fixed strength levels. The RF achieved high balanced accuracies across all knee extensor strength conditions, with mean values between 0.946 and 0.979. Peak performance was obtained at intermediate strength levels, particularly at 100 ± 5 N (mean = 0.979) and 150 ± 5 N (mean = 0.978). Even at the lowest and highest knee extensor strength, the classifier maintained strong performance (≥ 0.95 mean balanced accuracy). Fold-to-fold variability remained low, indicating that angle-related patterns are robustly captured by the RF model across a wide range of knee extensor strength magnitudes. The RF model generalizes well to knee extensor strength discrimination at fixed knee joint angles and to angle discrimination at fixed knee extensor strength levels. While both tasks yield high balanced accuracies, angle classification at fixed strength levels shows slightly higher and more uniform performance overall. This suggests that, under the present conditions, angular variations may be more consistently encoded in the features of the impedance measurements than knee extensor strength variations when the complementary variable is constrained.

**Table 3:** Balanced accuracy for evaluating the seven different force targets 50 N to 200 N in 25 N steps, including all ± 5 N samples, across the folds for specific fixed angles. The RF model with the tuned parameters was used

Balanced accuracy score				
Fold	20 ± 1°	40 ± 1°	60 ± 1°	80 ± 1°
1	0.891	0.941	0.984	0.921
2	0.914	0.848	0.905	0.923
3	0.952	0.894	0.917	0.890
4	0.939	0.894	0.968	0.874
5	0.952	0.909	0.921	0.905
Mean	0.930	0.897	0.939	0.903

**Table 4:** Balanced accuracy for evaluating the four different angle targets 20° to 80° in 20° steps, including all ± 1° samples, across the folds for specific fixed forces labels. The RF model with the tuned parameters was used

Balanced accuracy score							
Fold	50 ± 5 *N*	75 ± 5 *N*	100 ± 5 *N*	125 ± 5 *N*	150 ± 5 *N*	175 ± 5 *N*	200 ± 5 *N*
1	0.987	0.968	0.982	0.956	0.988	0.955	0.979
2	0.937	0.977	0.977	0.964	0.979	0.925	0.947
3	0.963	0.972	0.986	0.964	0.979	0.935	0.973
4	0.913	0.981	0.977	0.980	0.963	0.950	0.952
5	0.950	0.936	0.973	0.976	0.979	0.965	0.952
Mean	0.950	0.967	0.979	0.968	0.978	0.946	0.961

## Discussion

The aim of this study was to assess whether EIT, combined with a machine learning approach, can estimate dynamic knee joint angles and knee extensor strength during concentric contraction at a constant angular velocity. The results indicate that although RF performs best during validation, none of the evaluated models generalize well to data from unseen participants. This points to potential limitations in feature representation, dataset size, or class separability. Future work should therefore focus on improved regularization, feature engineering, or alternative modeling strategies to close the validation–test performance gap and enhance robustness.

One key reason for the performance difference between validation on a known participant and testing on an unseen individual is the significant variation in muscle and fat composition between individuals, which has a greater influence on the EIT data than the effect of knee extensor strength and the knee joint angle within an individual. The influence of individual body composition could be minimized by a higher number of subjects. Another reason for this is likely related to the intrinsic sensitivity of EIT to measurement noise and mechanical disturbances of the electrode belt. In our experiments, a single belt size was used while thigh circumferences varied, and concentric knee movements prevented continuous, uniform tension across all electrodes. These factors, together with the limited number of recordings, amplified inter-participant variability and complicated model training.

Analyses of knee extension angle and extensor muscle strength revealed that each parameter independently affects the EIT measurements. This can be explained by the fact that, with increasing knee extension, the muscle fibre length, pennation angle, and moment arm change, which modifies the effective cross-sectional area and the distribution of intracellular and extracellular fluid [31]. Consequently, the same level of neural activation produce different EIT signatures at different joint positions. The torque a muscle must generate to hold a given extension also depends on the joint geometry, so the muscle may need to exert more or less force to achieve the same rotational position, further altering the impedance signal. At a constant joint angle, higher knee extension moments lead to increased muscle thickness and tendon elongation, which likewise modifies the cross-sectional area.

The small sample size also limited our ability to capture the full spectrum of variations in belt tightness, thigh circumference, and tissue composition. This aligns with findings reported by [14], who also mentioned the need for larger, more diverse datasets to achieve robust generalization. In Liang, et al. [19] they report that if the muscle is stretched, the composition of the observed cross-section changes significantly, which leads to variations of the bioimpedance, and consequently the changes measured by EIT. Nevertheless, this study constitutes a first attempt to apply EIT for knee extensor strength estimation during concentric contractions rather than isometric measurements because dynamic strength assessments better reflect muscle function during daily-life activities [20, 21].

For the first proof of concept, a concentric contraction with one angular velocity was used. Future investigations should also assess correlations across different angular velocities, different modes of muscle contraction, including eccentric, isometric, and isokinetic contractions. Liang et al. [19] reported no association between EIT measurements and knee extensor strength during isometric contraction, whereas our findings revealed a significant correlation during concentric contraction. This discrepancy may be explained by the dynamic knee joint angle changes and muscle shortening that occur during concentric contraction, which are absent in isometric conditions.

Another limitation of this study is that the influence of antagonist muscles was not considered. Co-activation of the hamstrings could have occurred during the task to stabilize the knee joint, generating antagonistic moments that partially oppose the knee extensors [32]. Consequently, the actual muscle strength of the agonists may have been underestimated. Future studies should therefore investigate the influence of antagonist activity on knee extensor function and its impact on EIT data, for example by incorporating additional electromyographic (EMG) measurements.

Despite these challenges, the results provide insight into future directions. A valuable next step would be to acquire different contraction conditions, angle velocities, and multiple measurement sessions for each participant on different days. An evaluation of the model on data from different days would allow tracking true physiological changes from day-to-day measurement, thereby assessing inter-day generalization. Demonstrating reliable predictions across sessions would markedly increase the clinical relevance of EIT-based knee extension angle and knee extensor muscle strength estimation, enabling longitudinal monitoring of rehabilitation progress. In the future, EIT may not only provide a method to estimate muscle function under standardized dynamic conditions, but also offer potential for non-invasive, real-time monitoring of neuromuscular performance during daily-life activities. However, further research is required to better understand the relationship between muscle function and EIT-derived signals and to optimize EIT measurement protocols. To reduce inter-subject variability, the acquisition protocol should be expanded to include individual participant-specific parameters, such as tissue thickness, and multiple belt sizes should be fabricated to accommodate the range of thigh circumferences. Controlling electrode pressure and ensuring uniform skin contact should further improve signal quality. This approach would help to disentangle true physiological variability from measurement variance introduced by the EIT belt positioning, skin-electrode contact, and other day-to-day artifacts. Increasing the number of repetitions per participant would also provide a richer dataset for statistical analysis and model training. Disturbances and motion-induced artifacts increased noise in the measured raw data and complicated model training, despite the controlled laboratory environment [8].

In summary, our findings demonstrate that EIT measurements have potential for estimating knee joint angle and knee extensor strength, and we have identified several ways for addressing the current methodological limitations. By implementing the proposed refinements- standardized electrode belts, expanded subject-specific data collection, increased sample size, advanced subject-independent modeling techniques, additional evaluation of other contraction types and speeds, and inclusion of co-activation of other muscle groups- EIT may evolve into a reliable, non-invasive modality for continuous monitoring of musculoskeletal function, thereby supporting its eventual incorporation into clinical rehabilitation practice.

## Data Availability

The code is available on GitHub . The data presented in this study are available on reasonable request from the corresponding author. The data are not publicly available due to copyright issues.
