# Correction: Correlation study: electrical impedance-based approximation of knee joint angle and extensor strength during concentric knee extension

**DOI:** 10.1186/s12891-026-10024-7

**Published:** 2026-05-30

**Authors:** Jacob P. Thönes, Franziska Geiger, Judith Osterloh, Arash Keshavarz, Rainer Bader, Sascha Spors

**Affiliations:** 1 Institute of Communications Engineering, Rostock, Germany; 2 Department of Orthopaedics, Rostock University Medical Center, Rostock, Germany

**Correction: BMC Musculoskelet Disord 27**,** 177 (2026)**

**https://doi.org/10.1186/s12891-026-09634-y**

Following the publication of the original article [1], part of the article title is missing.

The complete article title is:

Correlation study: electrical impedance-based approximation of knee joint angle and extensor strength during concentric knee extension.

The old article title was:

Correlation study: electrical impedance-based approximation of knee joint angle and extensor.

This has been corrected above and the original article [1] has been updated.
